# Prophylactic Antibiotic-Loaded Cement, Absorbable and Nonabsorbable, for Implant-Based Breast Reconstruction

**DOI:** 10.1093/asj/sjaf198

**Published:** 2025-10-07

**Authors:** Alvin Nguyen, Garrison A Leach, Shahnur Ahmed, Robert C Clark, Sarah C Sorice, Rahim S Nazerali, Aladdin H Hassanein, Chris M Reid

## Abstract

**Background:**

Periprosthetic infection after postmastectomy breast reconstruction remains a significant issue and may result in explantation, with many patients choosing to forego reconstruction thereafter.

**Objectives:**

The aim was to evaluate the effectiveness of absorbable and nonabsorbable prophylactic antibiotic cement in reducing infection rates in implant-based breast reconstruction.

**Methods:**

A multi-institutional, retrospective review was performed of patients who underwent tissue expander (TE) reconstruction with permanent or absorbable antibiotic-loaded cement. Patients received either (1) nonabsorbable polymethylmethacrylate (PMMA) plates loaded with vancomycin and tobramycin or (2) absorbable calcium sulfate (CS) beads or discs loaded with gentamycin and vancomycin. Medical history, perioperative details, and complications were collected. Comparisons were made between those who received nonabsorbable PMMA and those who received absorbable CS.

**Results:**

A total of 295 TE reconstructions was performed on 189 patients. Ninety-three (31.5%) breasts received PMMA, and 202 (68.5%) received CS. Seven (2.4%) breasts had infections, with 1 (0.3%) occurring beyond 6 weeks postoperatively and 6 (2.0%) occurring within 6 weeks. Four infected breasts had previous debridement due to wound dehiscence or mastectomy flap necrosis, and 1 occurred within the setting of an infected drain site. One TE was lost from infection. Antibiotic-loaded, nonabsorbable PMMA and absorbable CS had no significant differences in infection rates (4.3% vs 1.5%, respectively). Patients did not receive prophylactic oral antibiotics.

**Conclusions:**

Antibiotic-loaded cement can be effective for infection prevention in implant-based reconstruction. Nonabsorbable PMMA and absorbable CS for local antibiotic delivery resulted in similar safety profiles. Oral prophylactic antibiotics can be omitted safely with utilization of local antibiotic delivery.

**Level of Evidence: 3 (Therapeutic):**

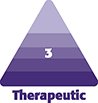

Tissue expander–based breast reconstruction continues to be the most common operative technique for postmastectomy restoration in the United States.^1,2^ This technique is often chosen for a variety of considerations, such as technical ease, better control over the prosthetic pocket, and the ability to offload pressure on ischemic skin. However, tissue expander–based breast reconstruction still has a high rate of periprosthetic infections, with reports ranging between 8% and 30%.^3-7^ Periprosthetic infections are problematic, impacting quality of life and increasing healthcare costs through additional hospital stays and surgical procedures. Infections often result in explantation, with as many as 60% of patients electing to abandon further surgery.^8,9^

Previously reported strategies to reduce the risk of infection include the “no-touch” technique and antibiotic irrigation of the breast pocket.^10-12^ However, most periprosthetic infections occur more than 30 days after reconstruction, a postoperative period during which these strategies cannot provide appropriate protection.^3,13,14^ The use of systemic, oral antibiotics has been described previously, but has not demonstrated significant benefit and may result in adverse side effects and selecting for more virulent pathogens.^15-17^

Utilization of prophylactic, local antibiotic delivery systems can provide proper therapeutic effects during the postoperative period while also avoiding systemic side effects.^18^ Although this technique has been popularized and widely described within other fields such as orthopedic surgery, it remains limited within plastic surgery.^19-28^ Nonetheless, local antibiotic delivery with either nonabsorbable polymethylmethacrylate (PMMA) or absorbable calcium sulfate (CS) has been growing within the realm of breast reconstruction, demonstrating significant decreases in infection rates.^24-29^ Nonabsorbable, antibiotic-loaded PMMA was initially placed for breast implant salvage before being offered as infection prophylaxis in implant-based breast reconstruction.^9,26,27,30^ However, limitations to PMMA include pain and the inability to utilize it for direct-to-implant approaches. Therefore biodegradable CS was introduced as another form of local antibiotic delivery.^28,29^

This study aims to add to the concept of prophylactic, local antibiotic delivery in breast reconstruction by comparing 2 eluting systems and describing the efficacy within various hospital settings and patients. The purpose of this multi-institutional study was to evaluate prophylactic antibiotic cement, both absorbable and nonabsorbable, in implant-based breast reconstruction.

## METHODS

### Study Population

Between 2021 and 2023, 2 surgeons from 2 institutions began offering prophylactic, antibiotic-loaded cement as part of their standard 2-stage breast reconstruction protocol. The antibiotic eluting devices included nonabsorbable PMMA and absorbable CS. After obtaining institutional review board approvals from Indiana University and UC San Diego and completing data use agreements, a retrospective review was performed of consecutive patients who underwent tissue expander (TE) reconstruction with prophylactic antibiotic-loaded cement from November 2021 to February 2025. Patient data were collected independently and completely deidentified for collaboration.

Data collected included demographics, medical history, operative details, and 90-day postoperative complications (hematoma, infection, wound dehiscence, mastectomy skin flap necrosis, return to the operating room, device loss). Hematomas were defined as unexpected sanguinous fluid collections that required additional drainage, either in clinic or the operating room (OR). Infections were defined as any clinical signs that required antibiotics with or without an operation. Wound dehiscence was defined as any incision that required dressing changes, debridement, or reclosure. Flap necrosis was defined as patients requiring debridement or return to the OR. Implant loss was defined as TE removal without replacement due to a complication.

### Technique

Plane of reconstruction and placement of drains were based on surgeon preference. A.H.H. performed prepectoral reconstruction with drain placement in all cases, and utilized absorbable CS beads.^25^ C.M.R. performed a few dual-plane subpectoral reconstructions early in practice and transitioned to only prepectoral. In addition, C.M.R. transitioned to a drainless tissue expansion approach as the case series progressed, and utilized nonabsorbable PMMA plates before transitioning to CS discs (Figure 1, Video).^27^ Nonetheless, plane of reconstruction and employment of drains did not alter the utilization of antibiotic-loaded cement. Nonabsorbable PMMA plates were loaded with vancomycin and tobramycin, and absorbable CS discs or beads were loaded with gentamycin and vancomycin.

**Figure 1.**
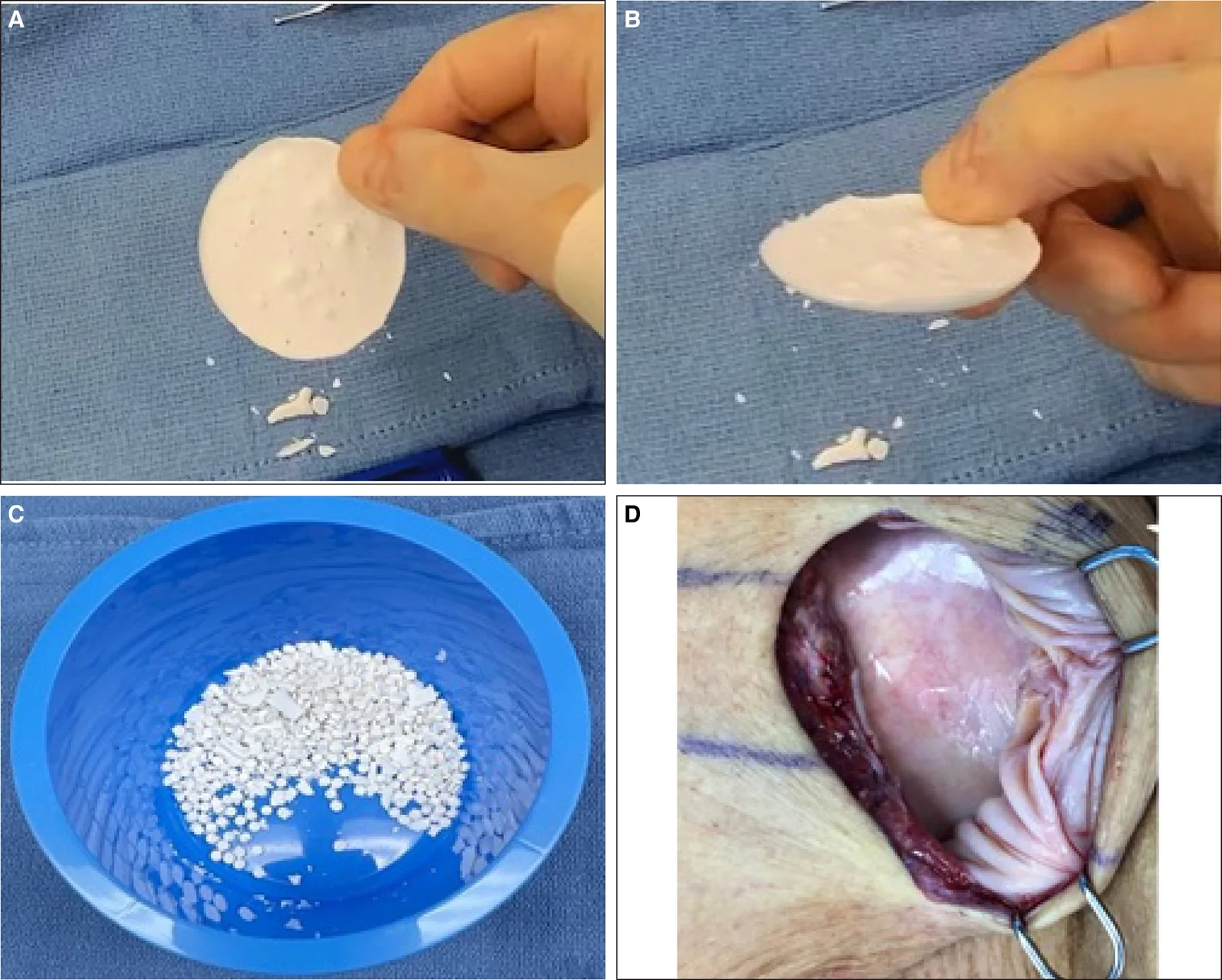
Subexpander implantation of absorbable antibiotic cement. (A, B) Antibiotic discs loaded with vancomycin and gentamycin. (C) Antibiotic cement prepared as beads. (D) Breast pocket after antibiotic cement has resorbed.

Antibiotic cement plates were prepared on a sterile back table intraoperatively, contoured to the patient's chest, and placed posterior to the expander. In cases that utilized antibiotic beads, the beads were placed both posterior to the expander over the pectoralis muscle and anterior to the TE. TEs were fastened in place with permanent sutures. Nonabsorbable PMMA plates were removed during the exchange for permanent implants or second-stage autologous reconstruction. Patients did not receive any antibiotics beyond a single perioperative dose and were not prescribed antibiotics upon discharge. Patients were informed that utilization of prophylactic, antibiotic-loaded PMMA and CS were not currently FDA indicated for breast reconstruction. Therefore employment of these antibiotic delivery devices for breast reconstruction was off-label.

### Statistical Analysis

All analyses were performed with R Statistical Software 4.4.1 (R, Vienna, Austria) with a *P* value < .05 considered statistically significant. Descriptive statistics were described with percentages, means, medians, standard deviations, and interquartile ranges (IQR). Comparisons were made between those who received nonabsorbable PMMA and those who received absorbable CS antibiotic cement at the time of TE placement. Normally distributed, continuous data were analyzed utilizing an unpaired *t* test. Asymmetrically distributed, continuous variables were analyzed by Wilcoxon rank-sum test. Categorical data were analyzed with a Pearson χ2 test or a Fisher's exact test. Multivariate regression analyses were performed to analyze whether cement type affected complication rates, with age, BMI, diabetes, smoking status, chemoradiation history, axillary node dissection history, laterality, and implant position included as covariates.

## RESULTS

### Demographics and Medical History

One hundred eighty-nine patients making up 295 breasts were included in the analysis (Table 1). A.H.H. provided 65 patients making up 118 breasts, and C.M.R. provided 124 patients making up 177 breasts. The average patient age was 48.7 years (SD 11.0 years), ranging from 24 to 78 years old, and average BMI was 27.1 kg/m^2^ (SD 5.7 kg/m^2^). Fifteen (7.9%) patients had a history of diabetes and 9 (4.8%) were current smokers. Eight (4.2%) patients underwent preoperative radiation and 54 (28.6%) had preoperative chemotherapy. Seventy-four (38.2%) patients underwent adjuvant radiation and 113 (59.8%) received postoperative chemotherapy. Forty-two (22.2%) patients underwent axillary lymph node dissection. Ninety-three (31.5%) breasts received antibiotic-loaded PMMA plates, and 202 (68.5%) breasts received absorbable CS antibiotic discs or beads. Ranging from 8 to 1065 days, median follow-up was 242 days (IQR 157-385 days) and average was 301 days.

**Table 1.** Patient Characteristics

	PMMA	Calcium sulfate	*P*
Patients	62 patients	127 patients	
Breasts	93 breasts	202 breasts	
Age (SD)	47.4 years (9.5)	49.4 years (11.6)	0.20
BMI (SD)	25.8 kg/m^2^ (4.5)	27.7 kg/m^2^ (6.2)	0.06
Diabetes	6 patients (9.7%)	9 patients (7.1%)	0.74
Current smoker	3 patients (4.8%)	6 patients (4.7%)	1.0
Neoadjuvant radiation	2 patients (3.2%)	6 patients (6.5%)	1.0
Neoadjuvant chemotherapy	21 patients (33.9%)	33 patients (26.0%)	0.34
Adjuvant radiation	27 patients (43.5%)	47 patients (37.0%)	0.48
Adjuvant chemotherapy	45 patients (72.6%)	68 patients (53.5%)	0.02^a^
Axillary dissection	20 patients (32.2%)	22 patients (17.3%)	0.03^a^
Immediate reconstruction	19 patients (30.6%)	24 patients (18.9%)	0.10

BMI, body mass index; PMMA, polymethylmethacrylate; SD, standard deviation.

^a^Statistically significant, *P* < .05.

### Complications

Twenty-two (11.6%) patients experienced a complication within 90 days of TE placement (Table 2). This included 2 (0.7%) breast hematomas, 3 (1.0%) breasts with wound dehiscence, 21 (7.1%) breasts with mastectomy flap necrosis, and 7 (2.4%) breast infections. Seventeen (9.0%) patients returned to the OR at least once due to these complications. These included wound dehiscence closure, excisional debridement of mastectomy flap necrosis, and, in cases of infection or tissue expander exposure, replacement of the tissue expander and antibiotic cement. Four (1.4%) breasts had TE explantation without replacement due to mastectomy flap necrosis (2), hematoma (1), and infection (1). Regarding the 7 breast infections, 3 had previous mastectomy flap necrosis that required excisional debridement and reclosure, 1 previously developed mastectomy flap necrosis and had poor wound healing, 1 had a previous wound dehiscence that required excision and reclosure, and 1 occurred in the setting of an infected drain site; the patient was noted to have erythema and edema in the axillary region (Table 3). Six (2.0%) breast infections occurred within 6 weeks of TE placement and 1 (0.3%) breast infection occurred beyond 6 weeks. Average timing of infections among the 7 cases was 37.3 days (SD 17.7 days).

**Table 2.** Complications

	PMMA	Calcium sulfate	*P*
Patients	62 patients	127 patients	
Breasts	93 breasts	202 breasts	
Hematoma	1 breast (1.1%)	1 breast (0.5%)	1.0
Wound dehiscence	3 breasts (3.2%)	0 breasts (0.0%)	0.03^a^
Mastectomy flap necrosis	13 breasts (13.9%)	8 breasts (4.0%)	0.004^a^
Infection	4 breasts (4.3%)	3 breasts (1.5%)	0.21
Return to OR	11 patients (17.7%)	6 patients (4.7%)	0.01^a^
Implant loss	1 breast (1.1%)	3 breasts (1.5%)	1.0

OR, operating room; PMMA, polymethylmethacrylate.

^a^Statistically significant, *P* < .05.

**Table 3.** Timeline and Clinical History of Periprosthetic Breast Infections

Infection	Postoperative timing	Clinical history
Infection 1: *Pseudomonas aeruginosa*	69 days	Antibiotic-loaded PMMA, immediate reconstruction, axillary lymph node dissection, neoadjuvant chemotherapy, adjuvant chemoradiation, underwent excisional debridement for mastectomy flap necrosis before infection occurrence, presented to ED with symptoms of infection (redness, erythema)
Infection 2: *Staphylococcus aureus*	32 days	Antibiotic-loaded PMMA, immediate reconstruction, neoadjuvant and adjuvant chemotherapy, underwent excisional debridement for mastectomy flap necrosis before infection occurrence (presented with redness)
Infection 3: *Staphylococcus aureus*	9 days	Antibiotic-loaded PMMA, immediate reconstruction, axillary lymph node dissection, adjuvant chemoradiation, noted to have an infection at the drain site
Infection 4: Penicillin G– resistant *Staphylococcus aureus*	41 days	Antibiotic-loaded PMMA, immediate reconstruction, neoadjuvant chemotherapy, adjuvant chemoradiation, underwent excision and reclosure of wound dehiscence before infection occurrence
Infection 5: *Staphylococcus lugdunensis*	32 days	Antibiotic-loaded CS, 7-day delayed reconstruction, axillary lymph node dissection, adjuvant chemoradiation
Infection 6: Penicillin G– resistant *Staphylococcus aureus*	40 days	Antibiotic-loaded CS, immediate reconstruction, adjuvant radiation, underwent excisional debridement for mastectomy flap necrosis before infection occurrence
Infection 7: *Klebsiella oxytoca*	38 days	Antibiotic-loaded CS, immediate reconstruction, developed marginal necrosis of mastectomy flaps, had poor wound healing, and went on to develop infection, expander removed without replacement

The average timing of infections among the 7 cases was 37.3 days (SD 17.7 days). CS, calcium sulfate; ED, emergency department; PMMA, polymethylmethacrylate; SD, standard deviation.

### Antibiotic Cement Comparisons

When compared to patients who received nonabsorbable PMMA plates, patients who received absorbable CS beads or discs had statistically significant lower rates of adjuvant chemotherapy (72.6% vs 53.5%, respectively; *P* = .02) and axillary lymph node dissection (32.2% vs 17.3%, respectively; *P* = .03) (Table 1). There were no statistically significant differences between the 2 groups regarding age, BMI, diabetes, current smoking status, neoadjuvant radiation, neoadjuvant chemotherapy, or adjuvant radiation. Compared to those who received PMMA, patients who received absorbable CS had statistically significant lower rates of wound dehiscence (3.2% vs 0.0%, respectively; *P* = .03), mastectomy flap necrosis (13.9% vs 4.0%, respectively; *P* = .004), and lower rates in which patients returned to the OR (17.7% vs 4.7%, respectively; *P* = .01) (Table 2). Multivariate regression analyses continued to show that patients who received PMMA had significantly higher rates of mastectomy flap necrosis (OR = 3.43, *P* = .047) and return to the OR (OR = 4.5, *P* = .01). There were no statistically significant differences between nonabsorbable PMMA and absorbable CS regarding rates of hematoma, infection, and implant loss.

## DISCUSSION

Periprosthetic infections are common and devastating complications encountered in implant-based breast reconstruction, with rates as high as 30%.^3-7^ However, growing reports of prophylactic, local antibiotic systems have demonstrated early success in addressing this problem.^24-28^ As part of the ongoing pursuit to improve surgical outcomes and minimize infections, this multi-institutional study serves as the first to investigate and compare the efficacy of both nonabsorbable PMMA and absorbable CS as prophylactic, local antibiotic delivery systems for TE-based reconstruction, across different practices.

Antibiotic-loaded PMMA has been previously described in the setting of TE salvage, demonstrating successful sterilization and allowing more patients to complete their final reconstruction.^9,30^ The efficacy of antibiotic-loaded PMMA for implant salvage eventually transitioned into teams offering the intervention as infection prophylaxis, resulting in significantly lower infection rates (4%-6%).^26,27,29^ Although there is limited discussion of it in breast reconstruction literature, biodegradable CS also has been described as an option for local antibiotic delivery, with infection rates dropping below 4% and explantation rates dropping to 1.5%.^24,25,28^ Absorbable CS has been described in the settings of high-risk prepectoral TE reconstruction as well as nonselectively for all immediate TE reconstructions, with infection rates of 3.2% and 1.6%, respectively.^25,28^ Absorbable CS also has been employed in breast reconstruction implant salvage in conjunction with negative pressure wound therapy with instillation.^31^

Before implementation of prophylactic, local antibiotics, A.H.H and C.M.R had institutional infection rates of 6.1% and 16.7%, respectively, for patients undergoing 2-stage breast reconstruction.^27,28^ Within this cohort, a total of 7 (2.4%) breasts experienced infections. In addition, there were no significant differences in infection rates between those receiving nonabsorbable PMMA (4.3%) and those receiving absorbable CS (1.5%). With rates comparable to previous descriptions, this study highlights the efficacy of both antibiotic delivery systems as infection prophylaxis for breast reconstruction.^24-28^ More importantly, infection incidence is significantly lower when compared to patients who did not receive prophylactic, local antibiotics.^3-7^

It is important to note that patients did not receive systemic antibiotic prophylaxis. Due to the high infection risks associated with TE-based reconstruction, postoperative prescriptions of oral antibiotics are prevalent among plastic surgeons.^32^ However, there have been conflicting data regarding the efficacy of this approach.^15^ A randomized controlled trial by Phillips et al demonstrated no significant differences in infection rates with extended antibiotic prophylaxis, and even found a higher likelihood of more severe infections in the prolonged antibiotic group.^33^ Similarly, in a propensity score–matched cohort accounting for comorbidities and treatment factors, systemic antibiotic prophylaxis following TE-based reconstruction did not result in improved infection rates.^34^ And although systemic antibiotic prophylaxis resulted in similar infection rates, there have been notable microbiology differences of the associated infections, leading to organisms that are more difficult to treat.^17,33^

Adverse reactions associated with local antibiotic delivery remain rare in the literature. Nephrotoxicity is currently the most common complication described and has occurred mainly in the setting of PMMA.^35-37^ Average rate of acute kidney injury was about 5% but has been noted in mostly older patients and those with baseline renal dysfunction.^38,39^ In addition to a case report of delayed-type hypersensitivity, other rare side effects include temporary liver dysfunction, bone marrow suppression, and allergic reactions to a specific antibiotic.^38,40^ With these reports coming from mostly orthopedic surgery literature, continued studies within the realm of breast reconstruction will help further characterize the complication profiles associated with antibiotic-loaded PMMA and CS. And although local antibiotic delivery can greatly reduce side effects associated with systemic antibiotic administration, patients still require careful monitoring for these potential complications.

Numerous studies have investigated the antibiotic elution profiles of PMMA and CS. The most popular antibiotic combination includes an aminoglycoside (tobramycin or gentamycin) with vancomycin.^37,41^ However, it is important to note that factors such as cement preparation, size, and composition along with antibiotic type, ratio, and concentration will influence elution characteristics.^42-48^ These factors should be taken into clinical consideration for optimal duration and concentration of elution.

Regarding the longevity of antibiotic release from PMMA and CS, in-vitro studies have shown antibiotic concentrations above the minimum inhibitory concentration (MIC) of common organisms for 20 to 42 days.^42,45,48-51^ Biospecimens, collected within a range of 6 to 36 weeks from patients with arthroplasty cement spacers, also demonstrated antibiotic concentrations well above the MIC.^20,21,52^ In addition, radiographic data obtained from patients undergoing joint arthroplasty showed complete resorption of antibiotic-loaded CS at around 6 weeks.^53-55^ Therefore, with most periprosthetic infections occurring more than 30 days after breast reconstruction, there is strong reason to believe that local antibiotic delivery with PMMA or CS may provide appropriate protection during this critical postoperative window.^3,13,14^ Further work is needed to understand how elution profiles may be impacted based upon drain use, different cement formulations, and beads vs disc construction.

Within our cohort, only 1 (0.3%) breast infection occurred beyond 6 weeks postoperatively, and occurred within the setting of previous excisional debridement for mastectomy flap necrosis. Four of the 6 breast infections that occurred less than 6 weeks postoperatively were in the settings of marginal mastectomy flap necrosis with poor wound healing, recent operative treatments for mastectomy flap necrosis and wound dehiscence, and an infected drain site. Therefore, there may be substantial grounds to attribute these infections to preceding complications that introduced new organisms rather than to the actual efficacy of antibiotic-loaded PMMA or CS. In addition, breast skin flaps that had significant signs of intraoperative skin ischemia, or presumed risk of the event, underwent interval-staged TE placement 1 to 2 weeks after mastectomy to allow the skin to demarcate before TE placement. This transition to early interval-staged TE reconstruction occurred as the case series progressed, potentially resulting in the significantly higher rates of mastectomy flap necrosis and return to the OR for patients who received antibiotic-loaded PMMA early in the study. Nonetheless, infections rates remained relatively low and did not significantly differ between the 2 antibiotic delivery systems.

Results of this study should be considered within the context of its limitations. Antibiotic concentrations in the systemic circulation and breast pocket were not measured. In addition, there was lack of a control group. Rather, overall infection rates were compared to previous literature reports and historical controls, with comparisons primarily focused between those receiving PMMA and those receiving CS. Given our smaller sample size and low infection rates, another limitation of our study is that it may be underpowered. Future studies with larger patient populations and continued use of antibiotic cement may provide a clearer understanding of the impact of different cement types on infection prevention. The utilization of drains and mastectomy operative details were not included in the comparative analysis. With these variables being surgeon-specific, comparative analysis may be confounded by unknown factors specific to the institution and result in biased interpretations. Comparative analysis, however, was focused on known risk factors for periprosthetic infections.^3,13,56-58^ Nonetheless, even within various institutional settings and surgeon-specific practices, there is strong evidence suggesting the efficacy of antibiotic-loaded PMMA and CS as infection prophylaxis. Future study directions include understanding antibiotic elution profiles within the breast pocket, duration of antibiotic coverage, and antibiotic cement–related complications.

## CONCLUSIONS

Prophylactic antibiotic-loaded cement is effective for infection prevention with implant-based reconstruction. Transitioning from nonabsorbable PMMA to absorbable calcium sulfate for local antibiotic delivery resulted in similar safety profiles, making it an attractive solution for direct-to-implant reconstruction as well. Oral prophylactic antibiotics can be omitted safely with utilization of local antibiotic delivery.
